# ECG Beats Classification Using Mixture of Features

**DOI:** 10.1155/2014/178436

**Published:** 2014-09-17

**Authors:** Manab Kumar Das, Samit Ari

**Affiliations:** Department of Electronics and Communication Engineering, National Institute of Technology, Rourkela, Orissa 769008, India

## Abstract

Classification of electrocardiogram (ECG) signals plays an important role in clinical diagnosis of heart disease. This paper proposes the design of an efficient system for classification of the normal beat (N), ventricular ectopic beat (V), supraventricular ectopic beat (S), fusion beat (F), and unknown beat (Q) using a mixture of features. In this paper, two different feature extraction methods are proposed for classification of ECG beats: (i) S-transform based features along with temporal features and (ii) mixture of ST and WT based features along with temporal features. The extracted feature set is independently classified using multilayer perceptron neural network (MLPNN). The performances are evaluated on several normal and abnormal ECG signals from 44 recordings of the MIT-BIH arrhythmia database. In this work, the performances of three feature extraction techniques with MLP-NN classifier are compared using five classes of ECG beat recommended by AAMI (Association for the Advancement of Medical Instrumentation) standards. The average sensitivity performances of the proposed feature extraction technique for N, S, F, V, and Q are 95.70%, 78.05%, 49.60%, 89.68%, and 33.89%, respectively. The experimental results demonstrate that the proposed feature extraction techniques show better performances compared to other existing features extraction techniques.

## 1. Introduction

Electrocardiogram (ECG) signal which is the recording of the cardiac electrical activity provides the important information about heart's condition. Detection of ECG arrhythmias is necessary for the treatment of patients for diagnosing the heart disease at the early stage. It is very difficult for doctors to analyze long ECG records in the short period of time and also human eye is poorly suited to detect the morphological variation of ECG signal, hence imposing the need for an effective computer aided diagnostic (CAD) system. The automatic ECG signal analysis faces a difficult problem due to large variation in morphological and temporal characteristics of ECG waveforms of different patients as well as the same patients [1]. The ECG waveforms may differ for the same patient at different time and may be similar for different patients having different types of beats. For this reason, most of the ECG beats classification methods perform well on the training data but provide poor performance on the ECG waveforms of different patients.

In the last decade, a number of researchers have reported the different automatic ECG classification techniques [2–8]. An efficient system for recognition of the premature ventricular contraction from the normal beats and other heart diseases is reported in [2] and achieved accuracy of 97.14% using twelve files from MIT-BIH database for ECG classification. The ECG beat classification system based on higher order statistics of subband components and a feed forward back propagation neural network is described in the literature [3] and achieved the classification accuracy of 96.34%. In [4], a combination method based on the complementary features of mixture of experts and negative correlation learning methods is introduced for classifying the normal heartbeats, premature ventricular contraction (PVC) arrhythmias, and other abnormalities and achieved accuracy of 96.02%. The multilayer perceptron neural network classifier is used to classify the four types of ECG beats (normal beat, congestive heart failure beat, ventricular tachyarrhythmia beat, and atrial fibrillation beat) using Lyapunov exponents, wavelet coefficients, and the power levels of power spectral density (PSD) values obtained by eigenvector methods of the ECG signals as feature set and achieved average accuracy of 93.89% [5]. In [6], six types of beats including normal beat, PVC, fusion of ventricular and normal beat, atrial premature beat (A), right bundle branch block beat, and fusion of paced and normal beat obtained from the MIT-BIH arrhythmia database, are classified using particle swarm optimization and radial basis function neural network. Two classifiers are combined together using mixture of experts where the local classifier requires a cardiologist to annotate a segment of patient specific ECG and achieved accuracy of 94.0% for distinguishing the two classes using mixture of expert classifiers. In [7], the morphological and temporal features are extracted to classify the five classes of ECG signal using linear discriminant classifier and achieved lower average accuracy of 85.9%. The disadvantage of de Chazal et al. [7] method is that the fixed classification method does not take any variation in ECG pattern caused by personal or environmental differences. In [8], Jiang and Kong have used the Hermite transform coefficients and time intervals between two neighboring R-peaks of ECG signals based features and block based neural network as a classifier and classified five types of ECG beat with an accuracy of 96.6%. In this method, there are around 1015 parameters/thresholds which are set empirically with respect to the dataset used. Another problem of this method is that the reported block-based neural networks (BbNN) require equal sizes for input and output layers.

However, all aforementioned techniques have following drawbacks as follows. (i) In general, all these methods have not performed well due to their inconsistent performance when classifying new patients ECG waveform. (ii) Most of these techniques were tested only on limited ECG data base. (iii) Despite many ECG classification methods offered in the earlier literature, only few have employed a standard classification scheme for ECG arrhythmia such as ANSI/AAMI EC57:1998 [10]. (iv) Most of them use either time or frequency domain representation of the ECG signals as features though both time and frequency are equally important to consider morphological variation of ECG beats.

In this paper, a novel approach is proposed to patient adaptation while avoiding the above limitations. ECG classification strongly depends on extraction of features from ECG waveforms. In this work, significant features are extracted using S-transform (ST) and wavelet transform (WT) due to their time-frequency localization properties [11]. The ST has unique properties such as (i) frequency invariant amplitude response, (ii) progressive resolution, and (iii) absolutely referenced phase information which means that the phase information given by the ST refers to the argument of the cosinusoid at zero time [11]. Besides, the interpretation of the important signal information in the ST is apparent which will be beneficial to extract the important features from the ECG signal [12]. On the other hand, WT is an efficient tool for analyzing nonstationary ECG signals. It is also used to decompose an ECG signal which effectively isolates the relevant properties of the ECG signal morphology from the noise, baseline drift, and amplitude variation of the ECG signal [9]. Earlier researcher has used the WT coefficients at the appropriate scales as morphological feature vectors rather than the original signal time series and achieved good classification performance [9]. Therefore, the two feature extraction techniques are combined depending on their properties and advantages which could help to select the features more effectively than using them independently. MLP-NN is normally used as a classifier to discriminate the ECG signal using these features. In this paper, we have used the MLP-NN classifier because (i) it can be used to generate likelihood-like scores that are discriminative in the state level, (ii) it can be easily applied in hardware platform for its simple structure, (iii) it has the ability to approximate functions and automatic similarity based generalization property, and (iv) complex class distributed features can be easily mapped by neural network [13]. The proposed methods classify the five classes of ECG beat recommended by the AAMI standard and experimental results are compared with the other existing feature extraction techniques. This paper proposes effective feature extraction methods which exhibit highest ECG classification sensitivity for large database.

The remainder of this paper is structured as follows.  Section 2 presents the database used in this context. The proposed framework is described in Section 3. Experimental design is described in Section 4. Performance results and discussion are explained in details in Section 5 and Section 6. Finally, the conclusions of the paper are reported in Section 7.

## 2. Data Base

**Figure 1:**
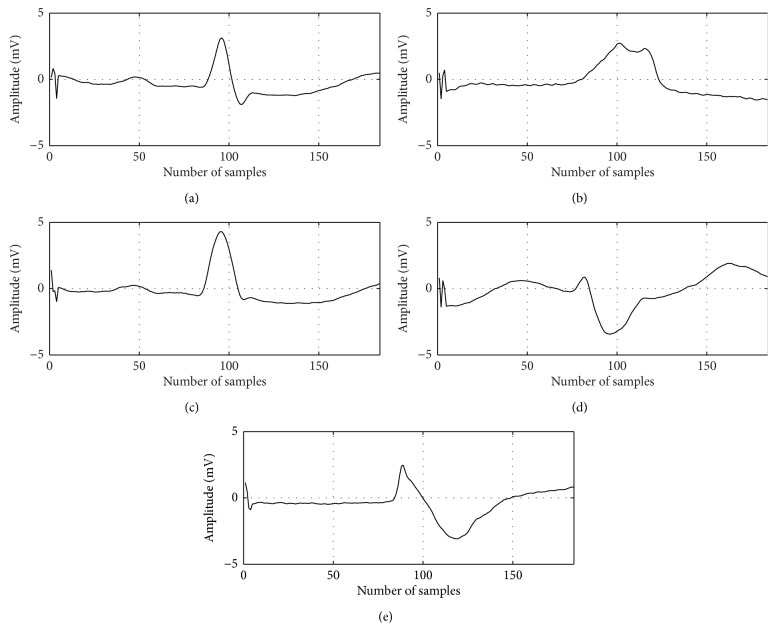
Five classes of ECG beat (a) normal (N), (b) supraventricular ectopic (S), (c) fusion (F), (d) ventricular ectopic (V), and (e) unknown (Q).

In this study, ECG data of MIT-BIH arrhythmia data base [14] are used for performance evaluation of the proposed ECG beat classification technique. This database contains 48 ECG recordings, each containing 30 min segment selected from 24 hrs recordings of 48 individuals. Each ECG signal is passed through a band pass filter at 0.1–100 Hz and sampled at 360 Hz. The 44 records from MIT-BIH arrhythmia database are used for performance assessment. According to the AAMI recommended practice the 4 paced beats are excluded in this experimental evaluation process because these beats do not retain sufficient signal quality for reliable processing [10]. This database contains different types of arrhythmias. In this paper, the normal and arrhythmia beats are combined based on AAMI standard which is described in Table 1. The AAMI convention is used to combine the beats into five classes of interest [7]: normal beat, left bundle branch block (LBBB), right bundle branch block (RBBB), and atrial escape and nodal junction escape beats belong to class N category; class V contains premature ventricular contraction (PVC) and ventricular escape beats, class S contains atrial premature (AP), aberrated premature (aAP), nodal junction premature (NP), and supraventricular premature (SP) beats, class F contains only fusion of ventricular and normal (fVN) beats, and class Q which is known as unknown beat contains paced beat (P), fusion of paced and normal (fPN) beats, and unclassified beats. Five classes of ECG arrhythmia are shown in Figure 1.

## 3. Proposed Framework

**Figure 2:**
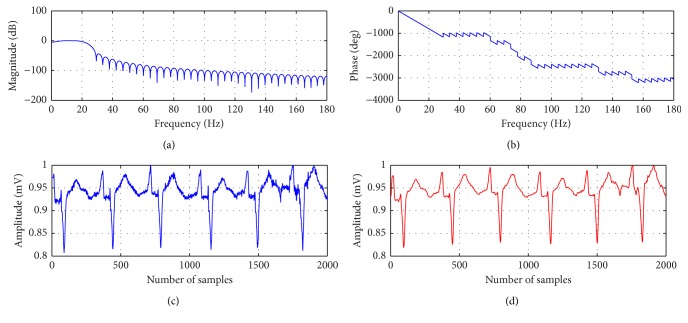
(a) Frequency spectrum of band pass filter; (b) phase spectrum of the filter; (c) ECG signal before applying the filter; (d) after applying the filter.

**Figure 3:**
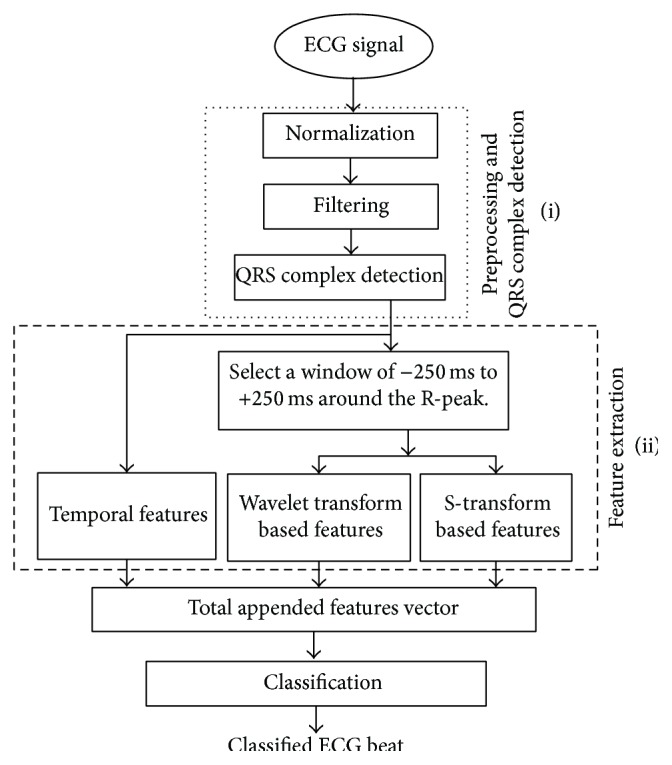
Block diagram of ECG classification using proposed hybrid feature extraction technique.

The block diagram of the ECG classification technique is shown in Figure 3. In this context, the proposed classification techniques consist of three main stages such as (i) preprocessing and QRS detection, (ii) feature extraction, and (iii) classifier. The preprocessing stage involves the following two substages: (i) normalize the amplitude of ECG signals to zero mean; this reduces the DC offset and eliminates the amplitude variance file to file; (ii) the bandpass filter (3–20 Hz) is used to contain most of QRS complex energy and least amount of high frequency noise and low-frequency baseline wander. Figures 2(a) and 2(b) show the frequency and phase spectrum of bandpass filter, respectively, and Figures 2(c) and 2(d) show the ECG signal before and after the use of bandpass filter. The proposed technique follows QRS complex detection as reported in Pan Tompkins' QRS detection algorithm [15]. This paper proposes feature extraction techniques based on ST and mixture of ST and WT based feature set.

### 3.1. Feature Extraction

Feature is a distinctive or characteristic measurement, transform, structural component extracted from a signal of a pattern. A feature extractor should reduce the pattern vector (i.e., the original waveform) to a lower dimension, which contains most of the important information from the original vector [16]. Generally, two types of features are extracted from one ECG cardiac cycle: (a) morphological features and (b) temporal features.

#### 3.1.1. Temporal Features

**Figure 4:**
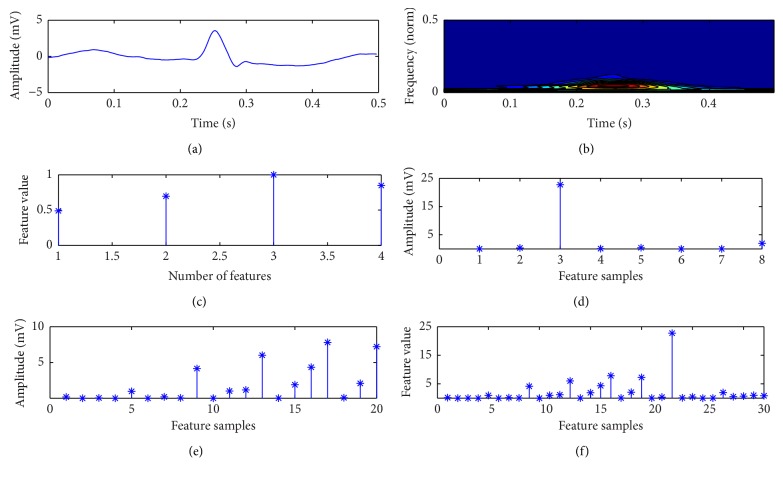
Extracted feature vector from one ECG cardiac cycle of Tape no. #200 ECG record. (a) ECG signal of selected samples; (b) ST of selected ECG signal; (c) extracted temporal feature set from selected ECG signal; (d) ST based morphological feature set; (e) WT based feature set; (f) combined set of WT, ST, and temporal features.

Four temporal features are extracted directly from RR-intervals of the preprocessed ECG signal. RR-intervals are calculated as the interval between successive heartbeats. The following are the four ways to extract the temporal features: (i) pre-RR-interval: RR-interval between a given heartbeat and the previous heartbeat; (ii) post-RR-intervals: the RR-interval between a given heartbeat and the following heartbeat; (iii) average RR-intervals: the mean of the RR-intervals for a recording and is considered as the same value for all heartbeats in a recording; (iv) local average RR-interval: averaging the RR-intervals of ten RR-intervals surrounding a heartbeat [7]. Thus, four temporal features are obtained from each ECG heart beat which is shown in Figure 4(c).

#### 3.1.2. Morphological Features

In this paper, three types of morphological features are used to classify the ECG beats: (a) ST based morphological features, (b) WT based morphological features, and (c) combined morphological feature of ST and WT. These features are extracted from one ECG cardiac signal. For morphological feature extraction, 180 samples are extracted from one ECG cardiac cycle by selecting a window of −250 ms to +250 ms around the R-peak.  Figure 4(a) indicates the time domain and time-frequency domain ECG signal, respectively.

(*1) Proposed ST Based Features*. The ST [11] is used to obtain the time-frequency representation of a time domain noisy ECG signal. The continuous ST *S*(*τ*, *f*) of a noisy ECG signal *h*(*t*) at time *t* = *τ* and frequency *f* is defined as

(1) $$\begin{array}{c} S\left(\tau ,f\right)=\overset{\infty }{\underset{-\infty }{\int }}h\left(t\right)\frac{\left|f\right|}{\sqrt{2\pi }}e^{-{\left(\tau -t\right)}^{2}f^{2}/2}e^{-i2\pi ft}dt. \end{array}$$

A voice *S*(*τ*, *f*_*o*_) is defined as a one-dimensional function of time for a constant frequency *f*_*o*_, which shows how the amplitude and phase for this exact frequency change over time. If the time series *h*(*t*) is windowed (or multiplied point by point) with a window function (Gaussian function) *g*(*t*), then the resulting spectrum is

(2) $$\begin{array}{c} H\left(f\right)=\overset{\infty }{\underset{-\infty }{\int }}h\left(t\right)g\left(t\right)e^{-i2\pi ft}dt, \end{array}$$

where generalized Gaussian function is

(3) $$\begin{array}{c} g\left(t\right)=\frac{1}{\sigma \sqrt{2\pi }}e^{-t^{2}/2\sigma^{2}} \end{array}$$

and then allowing the Gaussian to be a function of translation *τ* and dilation (or window width) *σ*,

(4) $$\begin{array}{c} S\left(\tau ,f,\sigma \right)=\overset{\infty }{\underset{-\infty }{\int }}h\left(t\right)\frac{1}{\sigma \sqrt{2\pi }}e^{-{\left(t-\tau \right)}^{2}/2\sigma^{2}}e^{-i2\pi ft}dt. \end{array}$$

This is a special case of the multiresolution Fourier transform because there are three independent variables in it; it is also impractical as a tool for analysis. Simplification can be achieved by adding the constraint restricting the width of the window to *σ* which is proportional to the period (or inverse of the frequency):

(5) $$\begin{array}{c} \sigma \left(f\right)=\frac{1}{\left|f\right|}. \end{array}$$

The reasons [17] for taking Gaussian window are as follows: (i) it is symmetric in time and frequency; the Fourier transform (FT) of a Gaussian is Gaussian, (ii) there are no side lobes in a Gaussian function, and (iii) it uniquely minimizes the quadratic time frequency moment about a time frequency point.

The Discrete ST [17] of the ECG signal *h*[*kT*] is given by

(6) $$\begin{array}{c} S\left[jT,\frac{n}{NT}\right]=\overset{N-1}{\underset{m=0}{\sum }}H\left[\frac{m+n}{NT}\right]e^{-2\pi^{2}m^{2}/n^{2}}e^{i2\pi mj/N}, \end{array}$$

where *H*[*n*/*NT*] is the FT of the *N* point time series *h*[*kT*] and *j*, *m*, *n* = 0,1,…, (*N* − 1). The output of ST is a complex valued matrix whose rows indicate the frequency and column indicates the time. The ST-amplitude, which is used to analyze the ECG signal, is defined as

(7) $$\begin{array}{c} A\left(kt,f\right)=\left|S\left[kT,\frac{n}{NT}\right]\right|. \end{array}$$

The proposed feature extraction technique ST is applied to the selected portion of ECG signal. The ST output is denoted as ST-matrix. Features are extracted by applying standard statistical techniques to the contours of the ST-matrix as well as directly on the ST-matrix. These features are very useful for detection, classification, and quantification of relevant parameters of ECG signals. Eight features are extracted from the ST output, four from the time-frequency contour (TF-contour) and remaining four from the time maximum amplitude plot (TmA-plot) [18]. Total eight significant features are extracted below in two cases.

**Case 1 .**

Feature extraction from TF-contour:

1. S1: standard deviation of the TF-contour having the largest frequency amplitude of TF-contour;
2. S2: mean of contour having largest frequency amplitude of TF-contour;
3. S3: the energy of contour having largest frequency amplitude of TF-contour.

**Case 2 .**

Extraction of features from TmA-plot:

(4) S4: maximum value of TmA-plot;
(5) S5: minimum value of TmA-plot;
(6) S6: mean value of the TmA-plot;
(7) S7: standard deviation of TmA-plot;
(8) S8: maximum energy of TmA-plot.

**Figure 5:**
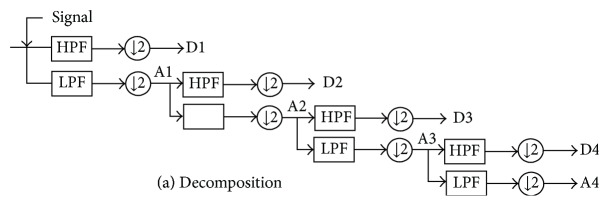
Subband decomposition of discrete wavelet transform.

Thus, eight morphological features are obtained by applying standard statistical techniques to the contours of the ST-matrix as well as directly on the ST-matrix which is shown in Figure 4(d).  Table 2 represents one way ANOVA results for different types of classes based on different types of features. If the *P* value (significance level) [19] in Table 2 is less than 0.05, there is a significant difference between the groups with a confidence level of 95%. This rule indicates that performances of S-transform based feature set of ECG signal are significantly different from each other.

(*2) WT Based Features*. The choice of appropriate wavelet and the number of decomposition levels are very important section on analysis of ECG signals using wavelet transform (WT) [20]. The decomposition levels are selected based on the maximum frequency components of the signal. The levels are taken such that those parts of the signal correlate well with the wavelet coefficients. In this paper, the number of decomposition levels is taken to be 4, i.e., ECG signals are decomposed into the details D1–D4 and one approximation coefficient A4 which is shown in Figure 5. The Daubechies wavelet of order 2 (db2) is chosen due to its similar morphological structure with the ECG signals [21]. The wavelet coefficients give a compact representation of the signal that indicates the distribution of signal energy in time and frequency. The computed detail and approximation wavelet coefficients of the ECG signals of each record are used as the feature vectors representing the ECG signal. For extracting the statistical features, 180 samples of ECG signal are taken from one ECG cardiac cycle by selecting a window of −250 ms to +250 ms around the R-peak. For each ECG beat, the detail coefficients at the first, second, third, and fourth levels (91, 47, 25, and 14 coefficients, resp.) and the approximation coefficients (14 coefficients) of fourth level decomposition are computed. Then, the total 191 wavelet coefficients are obtained from each ECG beat. In order to reduce the dimensionality of the extracted features, statistics over the set of the wavelet coefficients are used. The following statistical features are extracted using WT to represent the time-frequency distribution of the ECG signal:

1. F1: maximum value of the wavelet coefficients in each subband,
2. F2: mean value of the wavelet coefficients in each subband,
3. F3: minimum value of the wavelet coefficients in each subband,
4. F4: standard deviation of the wavelet coefficients in each subband,

where F1, F2, F3, and F4 are the feature of each subband. Therefore, 20 features are extracted from the selected subband of WT which is depicted in Figure 4(e).

(*3) Proposed Combined Features of ST and WT*. The proposed combined feature set is formed by appending the four temporal features, twenty WT based features, and eight ST based features.  Figure 4(f) represented the combined feature set of Tape no. #200 ECG record.

### 3.2. Classifier

The network topology is the MLP-NN classifier with a single hidden layer. A MLP-NN [22] is trained with the error back propagation algorithm. The input of MLP-NN is driven separately by the WT along with temporal based feature set [20] and proposed feature sets. The output layer has five neurons, which is equal to the number of ECG beat types to be classified. The number of input nodes is equal to the number of input features. The back propagation training with generalized delta learning rule is an iterative gradient algorithm designed to minimize the root mean square error between the actual output of a multilayered feed-forward, neural network and a desired output. Each layer is fully connected to the previous layer and has no other connection. The hyperbolic tangent function is applied as an activation function. The weight and bias values in the MLP-NN are updated with a learning rate of 0.5 which is chosen empirically. The smaller we make the learning rate parameter, the smaller the changes to the synaptic weights in the network will be from one iteration to the next and the smoother the trajectory in weight space will be. On the other hand, we make the learning rate parameter too large in order to speed up the rate of learning; the resulting large changes in the synaptic weights assume such a form that the network may become unstable (i.e., oscillatory). In order to achieve faster convergence with minimum oscillation, a momentum term may be added to the basic weight updating equation [22].

## 4. Experimental Design

**Figure 6:**
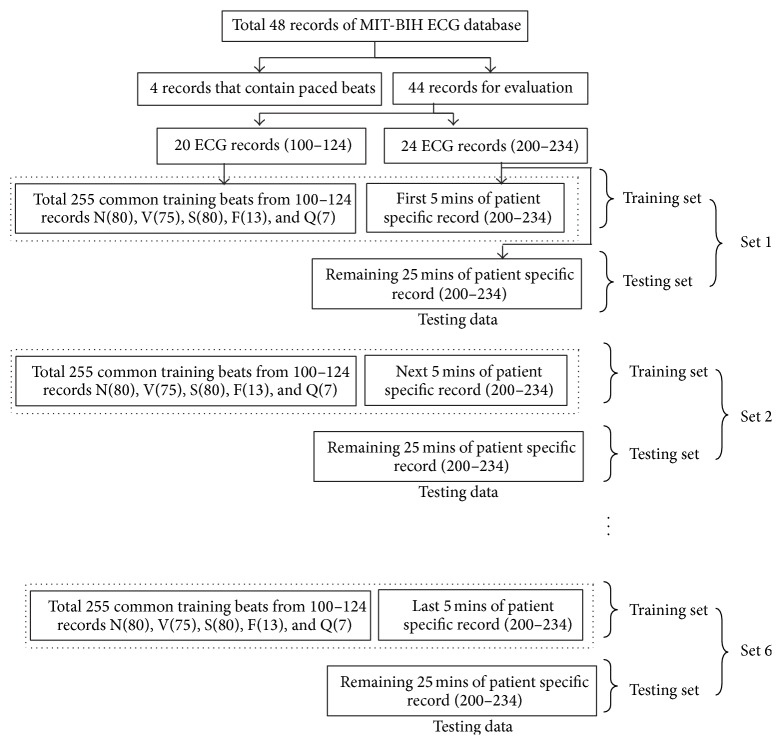
Diagram for 44 ECG recordings used as training and testing data set.

Classification experiments are performed using 44 records of the MIT-BIH arrhythmia database. In this work, a common training data set is constructed using first 20 records of MIT-BIH database (picked from the range 100–124, i.e., 100, 101, 103, 105, 106, 108, 109, 111, 112, 113, 114, 115, 116, 117, 118, 119, 121, 122, 123, and 124) which contains 80 from type-N, 75 from S, and 80 from V beat, and all (13) type F and all (7) type Q beats. In this technique, each record in the MIT-BIH data base is about 30 mins. Therefore, each record is subsampled into six sets. Each set contains 5 mins of ECG signal. Patient specific classifier is trained with a total of 255 common training beats and 5 mins of each patient specific record (picked from the range 200–234, i.e., 200, 201, 202, 203, 205, 207, 208, 209, 210, 212, 213, 214, 215, 219, 220, 221, 222, 223, 228, 230, 231, 232, 233, and 234). The remaining 25 mins of each record is used in testing purpose for classification evaluation. For second set of training, the classifier is trained with a total of 255 common training beats and next 5 mins of each patient specific record (picked from the range 200–234) and the remaining 25 mins of each corresponding record is used for testing. The process is repeated six times so that classifier is trained with 255 common training data plus 5 mins of each patient specific record and remaining 25 mins of each corresponding data is used for testing. The formation of each training and testing data set used for MLP-NN classifier is shown in Figure 6 using tree diagram.

## 5. Results

**Figure 7:**
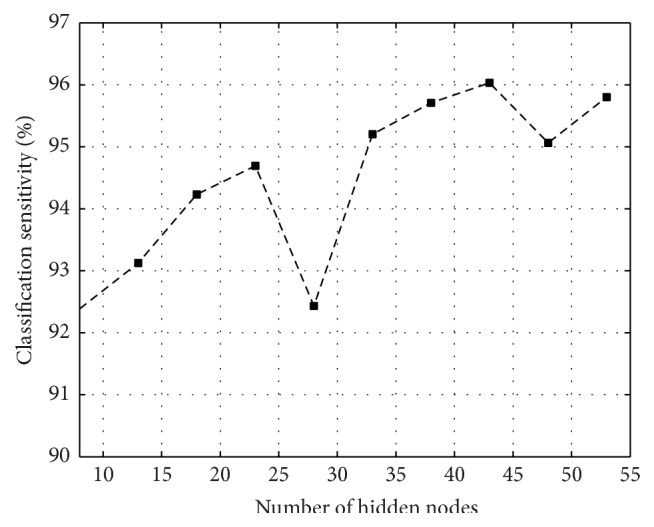
The performances of the proposed combined feature extraction method for Tape no. #200 ECG record when different hidden nodes are used.

The performance of the NN classifier mostly depends on the selection of hidden nodes. However, there are no such techniques to select the number of hidden nodes for better performance of the classifier. Hidden nodes in the hidden layer are selected empirically. As an example, for record no. 200, the variation of the classification performance with respect to hidden nodes is shown in Figure 7. In this study, 6-set cross validation techniques are used for training and testing of the MLP-NN classifier. The overall performance of the classifier is evaluated by taking the average of six sets. In this paper, classification performances are evaluated using three approaches. The first approach uses the WT based features combined with temporal features, the second approach uses the ST based features along with temporal features, whereas the third approach uses the mixture of ST and WT based features along with temporal feature set. The first method is indicated as WT based feature extraction method [20], second method is represented as Proposed-1 method, and third method is called Proposed-2 method. The correct classification or misclassification is quantified using four metrics such as True Positive (TP), True Negative (TN), False Positive (FP), and False Negative (FN). The classification sensitivity (Sen) and accuracy (Acc) are evaluated using these metrics. Classification accuracy is defined as the ratio of the number of correctly classified patterns (TP and TN) to the total number of patterns classified:

(8) $$\begin{array}{c} \text{Acc}=\frac{\text{TP}+\text{TN}}{\text{TN}+\text{FN}+\text{TP}+\text{FP}}. \end{array}$$

Sensitivity is the rate of correctly classified events among all events:

(9) $$\begin{array}{c} \text{Sen}=\frac{\text{TP}}{\text{TP}+\text{FN}}. \end{array}$$

**Figure 8:**
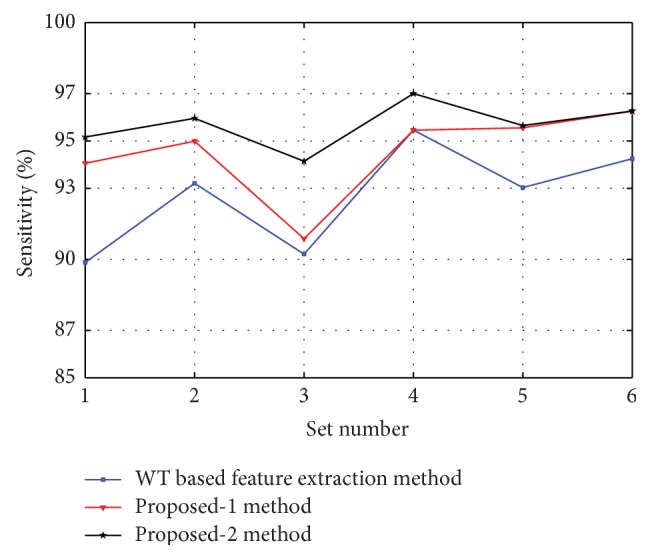
Sensitivity of N class using WT based, Proposed-1, and Proposed-2 feature extraction methods for six different sets used.

**Figure 9:**
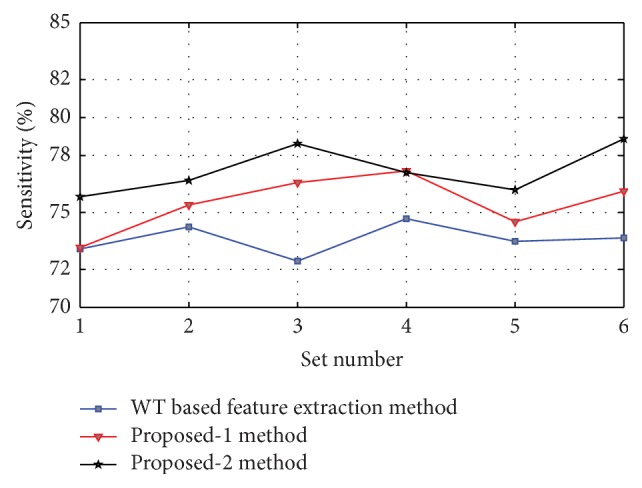
Sensitivity of S class using WT based, Proposed-1, and Proposed-2 feature extraction methods for six different sets used.

**Figure 10:**
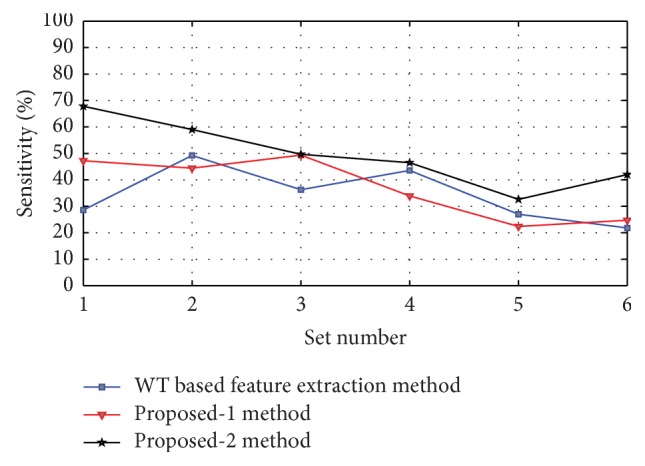
Sensitivity of F class using WT based, Proposed-1, and Proposed-2 feature extraction methods for six different sets used.

**Figure 11:**
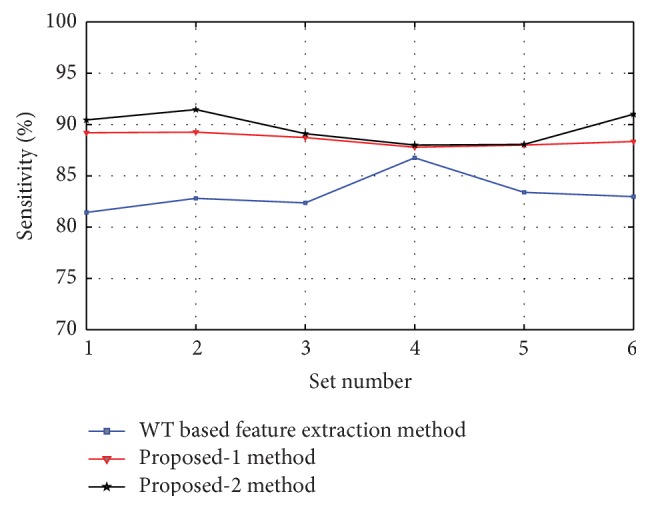
Sensitivity of V class using WT based, Proposed-1, and Proposed-2 feature extraction methods for six different sets used.

**Figure 12:**
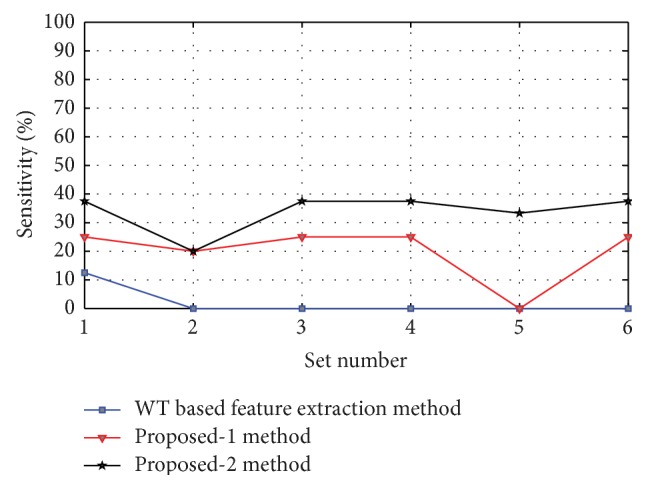
Sensitivity of Q class using WT based, Proposed-1, and Proposed-2 feature extraction methods for six different sets used.

**Figure 13:**
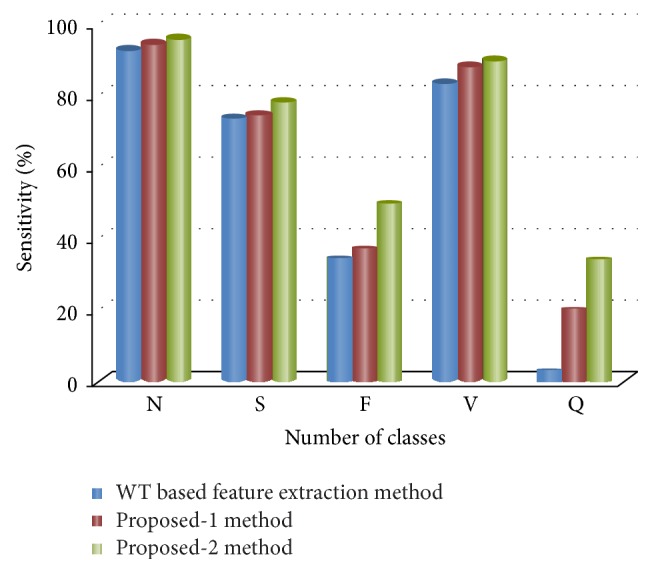
Bar diagram of all classes' sensitivity.

Figures 8, 9, 10, 11, and 12 show the sensitivity of N, S, F, V, and Q class during each set of the classification using WT based feature extraction method, Proposed-1 and Proposed-2 methods, respectively. It can be depicted from the figures that the Proposed-2 method shows the best performance compared the other techniques. The average performance from each of the classifiers is tabulated in Table 3. In WT based feature extraction method, the average sensitivity of N, S, F, V, and Q classes are 92.67%, 73.60%, 34.42%, 83.29%, and 2.08%, respectively, using 24 records whereas the Proposed-1 gives an average sensitivity of N, S, F, V, and Q class with 94.55%, 74.82%, 37.01%, 88.55%, and 20.00%, respectively, using the same records. On the other hand, the Proposed-2 shows an average sensitivity of N, S, F, V, and Q class with 95.70%, 78.05%, 49.60%, 89.68%, and 33.89%, respectively. The average sensitivity of all classes using three techniques is shown in Figure 13 using bar diagram. The average accuracy of N, S, F, V, and Q is 94.45%, 97.68%, 99.11%, 96.42%, and 99.63%, respectively, using Proposed-2 method whereas the Proposed-1 method yields that the accuracy of the N, S, F, V, and Q are 93.16%, 96.92%, 98.84%, 95.98%, and 99.58%, respectively. On the other hand, WT based feature extraction method provides an accuracy of N, S, F, V, and Q are 91.29%, 95.17%, 98.80%, 95.36%, and 99.49% respectively. The WT based feature extraction method [20] shows less average performance accuracy compared to Proposed-1 and Proposed-2 methods.

## 6. Discussion

This paper discusses the ECG beat classification using three feature extraction based techniques with MLP-NN classifier. From Table 3, it is seen that the sensitivities of F and Q are comparatively less than other classes because F beats are misclassified as N and V. F beats are difficult to distinguish from N and V beats because F beats are the union of ventricular and normal beats and their morphology and timing information closely resemble N and V beats [8]. The performances of the Proposed-1 and Proposed-2 methods in detecting class Q beats are worse because Q beats are less compared to other class in the training data and, hence, more Q beats are misclassified as other beats. On the other hand, the sensitivity of S class is less than the sensitivity of N class. The reason is that the QRS complex associated with an atrial premature beat in the S class has normal QRS duration and the same morphology as that of the sinus beat.  Table 4 yields a summary of studies on automated classification of ECG beats using the data obtained from MIT-BIH arrhythmia database. It is seen from the table that the average detection accuracy of the S-transform based feature extraction technique shows best performance compared to other existing techniques in the literature.

In this work, the classification performance is compared with WT based feature extraction method, Proposed-1 and Proposed-2 methods. The three different feature sets are applied separately on MLP-NN classifier. The WT based feature extraction method provides classification performance with average accuracy of 96.0% whereas Proposed-1 yields classification performance with 96.9% average accuracy. On the other hand, the Proposed-2 method shows the best performance with 97.5% average accuracy compared to other methods. The detection sensitivity of F and Q are comparably very less than the other classes. The reason for the worse classification performance in detecting the F and Q are that both classes are underrepresented in the training data, and, hence, more F and Q are misclassified as other classes. It is worthy that the less number of training beats are used for each patient's classifier which is approximately 2% of all beats in the training dataset. The proposed methods achieve better performances over other existing method for all the metrics used in five class detections. It is seen from Table 1 that the ECG arrhythmia is divided into five types of ECG class according to AAMI standards. For real time application, the proposed method detects the patient's arrhythmia as an AAMI class. For example, a patient has left bundle branch block (LBBB) arrhythmia but our algorithm detects it as AAMI N class.

**Figure 14:**
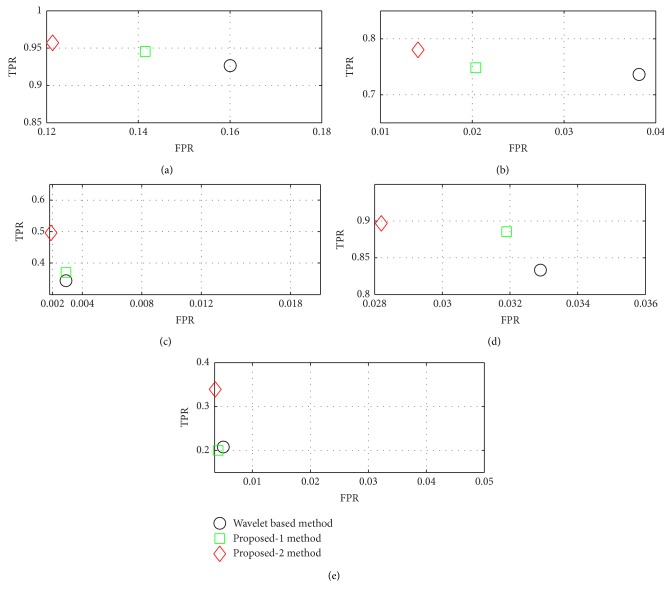
Comparison of true positive rate and false positive rate for three techniques in terms of (a) N class, (b) S class, (c) F class, (d) V class, and (e) Q class.

The relationship between sensitivity and specificity are described by the receiver operating characteristic (ROC) curve which alleviates improved analysis in terms of the classification performance of a diagnostic technique [8].  Figure 14 is marked as a ROC curve of N, S, F, V, and Q classes detection for 44 ECG records where the *x*-axis represents the false positive rate (FPR) and the *y*-axis represents the true positive rate (TPR). For accurate classification, TPR = 1 and FPR = 0 correspond to the upper left corner of the ROC curve. Therefore, the combination of TPR-FPR is considered better when it is more near to the upper left corner. It is observed that, for five types of AAMI class detection, proposed features provide higher TPR but lower FPR compared to wavelet based features.

## 7. Conclusion

In this work, ST based feature extraction and combination of ST and WT based feature extraction method are proposed separately to classify the ECG beats for each patient individually. In the proposed technique, the ST is effectively employed to extract the significant features which are combined with temporal features whereas the combined feature set is formed using the combination of WT, ST, and four temporal (pre-RR, post-RR, local RR, and avg RR) based features. The interpretation of the important signal information in the ST is apparent which will be beneficial to extract the important features from the ECG signal. On the other hand, WT is used to decompose an ECG signal according to the scale which effectively isolates the relevant properties of the ECG signal morphology from the noise, baseline drift, and amplitude variation of the ECG signal. Therefore, proposed feature extraction techniques enjoy the benefits of the above feature sets. These features are very useful for detection, classification, and quantification of relevant parameters of ECG signals. The performances of proposed features are compared with the other existing methods. Experimental results demonstrate that the proposed features provide better detection sensitivity than WT based features. The overall results of the proposed extracted feature methods also show an effective and efficient approach in computer-aided diagnosis of heart diseases based on ECG signals. The proposed system can be used as follows: (i) automated systems provide clinicians with the tools to be alerted in real time if life threatening conditions surface in their patients. As a result, automatic detection and classification of cardiac electrophysiology using biomedical signal processing techniques have become a critical aspect of clinical monitoring.

## Figures and Tables

**Table 1:** ECG class description using AAMI standard.

AAMI class	MIT-BIH heart beat types				
Normal beat (N)	Normal beat (N)	Left bundle branch block beat (L)	Right bundle branch block beat (R)	Atrial escape beat (e)	Nodal (junctional) escape beat (j)

Supraventricular ectopic beat (S)	Atrial premature beat (A)	Aberrated atrial premature beat (a)	Nodal (junctional) premature beat (J)	Supraventricular premature beat (S)	

Ventricular ectopic beat (V)	Premature ventricular contraction (V)	Ventricular escape beat (E)			

Fusion beat (F)	Fusion of ventricular and normal beat (F)				

Unknown beat (Q)	Paced beat (/)	Fusion of paced and normal beat (f)	Unclassified beat (Q)		

**Table 2:** One way ANOVA results for different types of classes.

Feature type	Source of variation	Sum of squares	df	Mean squares	*F*	*P*
S-transform	Between the groups	28.4927	−1	−28.4927	−0.49	<0.0001
	Within the groups	924.7985	16	57.7999		
	Total	**953.2912**	**15**			

**Table 3:** Performance comparison of the WT based feature extraction method, Proposed-1, and Proposed-2 methods using MIT-BIH database.

Method	N		S		F		V		Q	
	Acc	Sen	Acc	Sen	Acc	Sen	Acc	Sen	Acc	Sen
WT based feature extraction	91.29	92.67	95.17	73.60	98.80	34.42	95.36	83.29	99.49	2.08
Proposed-1	93.16	94.55	96.92	74.82	98.84	37.01	95.98	88.55	99.58	20.00
Proposed-2	94.45	95.70	97.68	78.05	99.11	49.60	96.42	89.68	99.63	33.89

**Table 4:** Summary of the studies on the different ECG classification technique using MIT-BIH database.

Literature	Features	Classifier	Classes	Accuracy
$\ddot{\text{U}}$beyli [5]	Lyapunov exponents and wavelet coefficients	ANN classifier	4	93.9
Kor$\ddot{\text{u}}$rek and Do$\overset{ˇ}{\text{g}}$an [6]	Temporal feature set	PSO and RBFNN	6	96.3
de Chazal and Reilly [7]	Morphology and heartbeat interval	Linear discriminant	5	85.9
Inan et al. [9]	WT + timing interval	ANN	3	95.2
Jiang and Kong [8]	Hermite transform coefficients and time intervals	Block based NN	5	96.6
Proposed-1	ST + Temporal	MLP-NN	5	96.9
Proposed-2	ST + WT + Temporal	MLP-NN	5	97.5
